# A Potential Late Stage Intermediate of Twin-Arginine Dependent Protein Translocation in *Escherichia coli*

**DOI:** 10.3389/fmicb.2019.01482

**Published:** 2019-07-11

**Authors:** Hendrik Geise, Eyleen Sabine Heidrich, Christoph Stefan Nikolin, Denise Mehner-Breitfeld, Thomas Brüser

**Affiliations:** Institute of Microbiology, Leibniz University Hannover, Hannover, Germany

**Keywords:** twin-arginine translocation, membrane protein complexes, protein translocation, *Escherichia coli*, photo cross-linking

## Abstract

The twin-arginine translocation (Tat) system transports folded proteins across membranes of prokaryotes, plant plastids, and some mitochondria. According to blue-native polyacrylamide gel electrophoresis after solubilization with digitonin, distinct interactions between the components TatA, TatB, and TatC result in two major TatBC-containing complexes in *Escherichia coli* that can bind protein substrates. We now report the first detection of a TatABC complex that likely represents the state at which transport occurs. This complex was initially found when the photo cross-linking amino acid *p*-benzoyl-l-phenylalanine (Bpa) was introduced at position I50 on the periplasmic side of the first trans-membrane domain of TatC. Cross-linking of TatC_I50Bpa_ resulted in TatC-TatC-cross-links, indicating a close proximity to neighboring TatC in the complex. However, the new complex was not caused by cross-links but rather by non-covalent side chain interactions, as it was also detectable without UV-cross-linking or with an I50Y exchange. The new complex did not contain any detectable substrate. It was slightly upshifted relative to previously reported substrate-containing TatABC complexes. In the absence of TatA, an inactive TatBC_I50Bpa_ complex was formed of the size of wild-type substrate-containing TatABC complexes, suggesting that TatB occupies TatA-binding sites at TatC_I50Bpa_. When substrate binding was abolished by point mutations, this TatBC_I50Bpa_ complex shifted analogously to active TatABC_I50Bpa_ complexes, indicating that a defect substrate-binding site further enhances TatB association to TatA-binding sites. Only TatA could shift the complex with an intact substrate-binding site, which explains the TatA requirement for substrate transport by TatABC systems.

## Introduction

The twin-arginine translocation (Tat) system transports folded proteins across the cytoplasmic membrane of prokaryotes, the thylakoid membrane of plant plastids, and the inner membrane in some mitochondria (Hou and Brüser, 2011; Hamsanathan and Musser, 2018; Petrů et al., 2018). The Tat-dependent translocation is driven by the membrane potential that is generated by ionic gradients at energy-transducing membranes, which is why this system is restricted to such membranes (Cline, 2015). In *Escherichia coli*, a fully functional Tat-translocon assembles from the three components TatA, TatB, and TatC (Sargent et al., 1999). A second paralog of TatA, TatE, can mix into these systems or substitute TatA, without being specifically required for the transport of a known Tat substrate (Sargent et al., 1999). TatA/E and TatB are evolutionary related and share the same principle structural organization in their functionally important N-terminal half (Yen et al., 2002; Hu et al., 2010; Zhang et al., 2014). They are N-terminally membrane anchored by a short hydrophobic region, followed by a hinge and an amphipathic helix on the cytoplasmic surface of the membrane. This amphipathic helix is followed by some negatively charged residues and regions that are not really conserved anymore between TatA/B family proteins (Hou and Brüser, 2011). TatC consists of six transmembrane helices (TM1–6) with cytoplasmic N- and C-termini and loops on the cytoplasmic and periplasmic sides of the membrane (Rollauer et al., 2012; Ramasamy et al., 2013). Tat substrates contain N-terminal signal peptides with the eponymous highly conserved twin-arginine motif, which is recognized by a binding site that is formed by the N-terminus and the first cytoplasmic loop of TatC (Ramasamy et al., 2013). TatC binds TatB and also TatA, and corresponding binding sites have been identified (Alcock et al., 2016). TatB and TatC interact tightly, and earlier studies with the homologous TatABC systems in plants and *E. coli* were suggestive for a substrate-induced recruitment of TatA to TatBC (Cline and Mori, 2001; Mori and Cline, 2002; Alami et al., 2003). While cross-linking and co-purification analyses later demonstrated that TatA also interacts with TatBC independently of substrate binding (Aldridge et al., 2014; Behrendt and Brüser, 2014), active transport apparently increases the affinity of TatA to TatBC, as reflected by a recruitment of TatA-XFP fusion proteins to TatBC in response to substrate overproduction (Alcock et al., 2013; Rose et al., 2013). Besides TatBC, also TatA interacts with the signal peptide of precursor proteins, but this interaction contributes to transport without being involved in the RR-motif recognition by TatBC (Taubert et al., 2015). The Tat complexes are usually analyzed by BN-PAGE in combination with Western-blotting or other labeling methods. Solubilized TatA tends to form multiple homooligomers that are visible as a dense ladder in BN-PAGE (Oates et al., 2005; Richter and Brüser, 2005). These highly abundant TatA homooligomers so far prevented the direct identification of TatA as constituent of any of the TatBC-containing complexes by BN-PAGE (Behrendt and Brüser, 2014). However, cross-linking experiments showed that TatA and TatB share the same binding sites of TatC, and positions of TatA and TatB are believed to switch in course of transport (Habersetzer et al., 2017). As TatA is required for Tat transport, it is important to reveal the complexes that contain all three components.

TatB and TatC have been detected in two substrate-free and two substrate-associated complexes in the range of 400–700 kDa (Behrendt and Brüser, 2014). As the migration behavior of solubilized membrane protein complexes in BN-PAGE is influenced by the detergent and lipid content of solubilized complexes, the BN-PAGE deduced molecular masses do not permit the estimation of individual subunit numbers. Some variation of reported Tat complex molecular masses in BN-PAGE analyses may have been caused by lot variations of the commonly used mild detergent digitonin, which is enriched from extractions of foxglove (*Digitalis purpurea*). For a clear assignment, it is therefore important to include the wild-type complexes in each study. To clarify the designation of Tat complexes, we now name the smaller substrate-free TatBC-containing complex of *E. coli* Tat-complex 1 (TC1; previously termed 370, 430, or 440 kDa complex; Oates et al., 2005; Richter and Brüser, 2005; Orriss et al., 2007; Huang et al., 2017) and the larger substrate-free TatBC-containing complex Tat-complex 2 (TC2; previously termed 580 kDa or “higher molecular weight variant” TatBC complex; Richter and Brüser, 2005; Huang et al., 2017). As mentioned above, both of these complexes can in principle also contain TatA, but the continuous dissociation of TatA from TatBC during solubilization and purification and the accompanying formation of homooligomeric TatA associations in a wide range of sizes prevented so far the assignment of TatA to these complexes. Both complexes are easily identified by BN-PAGE without overproduction of Tat substrates (Richter and Brüser, 2005; Behrendt and Brüser, 2014). Due to harsher BN-PAGE conditions, other groups detected TC2 only with transport-enhancing mutations (Huang et al., 2017). The corresponding substrate-bound shifted complexes, now termed TC1S or TC2S, can so far only be detected upon substrate overproduction (Behrendt and Brüser, 2014). To facilitate comparisons, we suggest the use of this nomenclature for future studies.

During characterizations of TatC variants with individual residues substituted by the artificial cross-linking amino acid *p*-benzoyl-L-phenylalanine (Bpa), we found with a TatC_I50Bpa_ mutation a new TatBC-containing complex larger than TC2S. The formation of this complex did not relate to a Bpa cross-link and depended on TatA. Mutational inactivation of the substrate-binding site permitted the formation of this complex in the absence of TatA, suggesting that TatB can be recruited to TatA-binding sites in such inactive complexes. The data indicate that subtle changes in the substrate-binding site can shift the TatBC complex to a higher associated state in the absence of TatA, but TatA is required for this shift when the substrate-binding site is functional. As the TatABC_I50Bpa_ system is active, and as the complex does not contain substrate anymore, it is likely that the described new complex represents a late translocation state, kinetically stabilized by the TatC_I50Bpa_ mutation.

## Materials and Methods

### Strains and Growth Conditions

The *tatABCDE* deficient *E. coli* strain DADE D6 ara^R^ (Lindenstrauss et al., 2010) was used for physiological and biochemical analyses, and *E. coli* XL1-Blue MRF’ Tet (Agilent) was used for cloning. Strains were grown aerobically at 37°C in LB medium [1% (w/v) tryptone, 1% (w/v) NaCl, 0.5% (w/v) yeast extract] in the presence of appropriate antibiotics (100 μg/ml ampicillin, 25 μg/ml chloramphenicol, and 12.5 μg/ml tetracycline) and harvested after 5 h. Cultures carrying pABS- or pDE-derived plasmids were normalized to an OD_600_ of 1.0 and cultures carrying pZX31-derived plasmids to an OD_600_ of 2.0. Cultures containing pBW-*efeB-strep* were harvested after 3 h growth with 0.05% (w/v) rhamnose added at an OD_600_ of 0.6. For incorporation of Bpa at amber stop codons in strains carrying the pEVOL-*p*BpF system, 100 μM Bpa was added simultaneously with 100 μM arabinose. For optional cross-linking, cultures were grown for 5 h before irradiation with UV light at 365 nm for 30 min at ambient temperature, normalization, and further processing.

### Plasmids and Genetic Methods

pEVOL-*p*BpF-tet, which encodes an orthogonal Bpa-specific suppressor tRNA/aminoacyl tRNA synthetase pair used for incorporation of Bpa at introduced amber stop codons, was donated by Peter G. Schultz (Young et al., 2010). The vector pDE-*tatABC-h6*, used for constitutive *tatABC* expression in pEVOL-*p*BpF-tet-containing strains, was generated by cloning the NheI-XbaI digested *ori* ColA from pCOLAduet-1 (Novagen) into the corresponding sites of pABS-*tatABC-h6*. pABS-*tatABC-h6* was generated by substituting *pspC* in pABS-*pspC-h6* (Mehner et al., 2012) with *tatABC*, using NdeI and BamHI restriction sites and the primer pair *tatA*-NdeI-F 5′-TCT TCT CAT ATG GGT GGT ATC AGT ATT TGG C-3′ and *tatC*-BamHI-R 5′-CAA GCG GAT CCT TCT TCA GTT TTT TCG CTT TCT GCT TC-3′. pABS-*tatABC-h6* results in constitutive P*_tatA_*-dependent expression of the *tatABC* genes, with TatC produced as C-terminally hexahistidine-tagged protein. The plasmid pZX31-*tatBC-h6*, used for constitutive *tatBC* expression, was generated by cloning *ori* ColA into AvrII-SpeI digested pZA31-*tatBC-h6* using the primer pair ColA-AvrII-F 5′-GAT CCC TAG GAA ACG TCC TAG AAG ATG CCA GGA GGA TA-3′ and ColA-SpeI-R 5′-GAT CAC TAG TTG GTG TCG GGA ATC CGT AAA GG-3′. For generation of pZA31-*tatBC-h6*, *tatBC* was amplified using the primer pair *tatB-*EcoRI-F 5′-GAA GAC GCG AAT TCC CAC GAT AAA GAG C-3′/*tatC-h6*-PstI-R 5′-TAG CCA CTG CAG TTA ATG GTG ATG GTG ATG GTG TTC TTC AGT TTT TTC GCT TTC-3′ and cloned into the corresponding sites of pZA31MCS (Expressys, Bammental). Single amino acid exchanges in TatC were introduced by QuikChange™ mutagenesis (Stratagene) of pDE-*tatABC-h6* or pZX31-*tatBC-h6*, using the forward primers *tatC*-A47Bpa-F 5′-CAT CTA TCA CCT GGT ATC CTA GCC ATT GAT CAA GCA GTT G-3′, *tatC*-P48Bpa-F 5′-CTA TCA CCT GGT ATC CGC GTA GTT GAT CAA GCA GTT GCC G-3′, *tatC*-L49Bpa-F 5′-GGT ATC CGC GCC ATA GAT CAA GCA GTT GC-3′, *tatC*-I50Bpa-F 5′-CAC CTG GTA TCC GCG CCA TTG TAG AAG CAG TTG CCG CAA GGT TC-3′, *tatC*-I50Y-F 5′-GGT ATC CGC GCC ATT GTA TAA GCA GTT GCC GCA AG-3′, *tatC*-I50F-F 5′-GTA TCC GCG CCA TTG TTC AAG CAG TTG CCG-3′, *tatC*-I50W-F GTA TCC GCG CCA TTG TGG AAG CAG TTG CCG CAA G-3′, *tatC*-K51Bpa-F 5′-CCG CGC CAT TGA TCT AGC AGT TGC CGC AAG-3′, *tatC*-Q52Bpa-F 5′-GCG CCA TTG ATC AAG TAG TTG CCG CAA GGT TC-3′, *tatC*-F94Q-F 5′-CTA TCA GGT GTG GGC ACA GAT CGC CCC AGC GCT G-3′, and *tatC*-E103A-F 5′-CGC TGT ATA AGC ATG CGC GTC GCC TGG TGG TG-3′ in conjunction with reverse primers that cover the identical sequence region. The vector pBW-*ycdB-strep* (Sturm et al., 2006) was used for the overexpression of *efeB* (formerly called *ycdB*). All genetic constructs were confirmed by sequencing.

### Biochemical Methods

BN-PAGE was performed as described previously (Richter and Brüser, 2005), but without *β*-mercaptoethanol in the buffers. TatABC complexes were solubilized with 1% digitonin and purified by Ni-NTA affinity chromatography using 50 mM Bis-Tris pH 7.0, 20% (w/v) sucrose, 10 mM MgCl_2_, 0.1% digitonin as buffer system, and 250 mM imidazole containing buffer for elution. When indicated, an additional size exclusion chromatography purification step was applied using the same buffer. Sodium dodecyl sulfate-polyacrylamide gel electrophoresis (SDS-PAGE) analysis was carried out by standard methods (Laemmli, 1970). For immunoblots, proteins were semi-dry blotted on nitrocellulose membranes and blots were developed using antibodies directed against synthetic C-terminal peptides of TatA, TatB, TatC, or purified EfeB, using the ECL system (GE Healthcare) for signal detection. HRP-conjugated goat anti rabbit anti-bodies (Roth) served as secondary antibodies. For stripping, blots were incubated with 100 mM glycine pH 2.8, 1% SDS, 0.2% Tween 20 for 30 min, followed by three washing steps with PBS and a second blocking (PBS with 5% skim milk). Stripped blots were again incubated with the secondary antibody and developed to ensure that they gave no detectable background and thereafter used for new detections. The chain formation phenotype was assessed by phase contrast microscopy. SDS sensitivity was determined by aerobic growth in LB medium containing 4% (w/v) sodium dodecyl sulfate (SDS), using the quotient of the OD_600_ with/without SDS after 3 h of growth (Ize et al., 2003).

## Results

### The Region Around Position I50 of TatC Contacts Neighboring TatC Protomers

**Figure 1:**
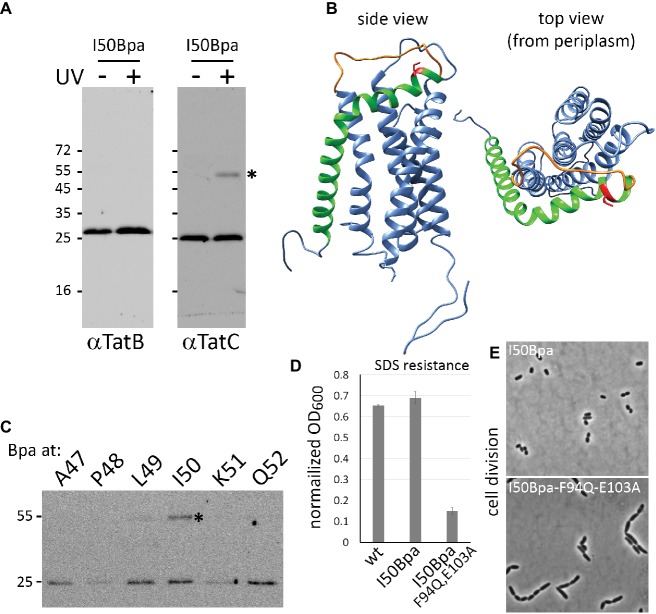
TatC_I50Bpa_ is functional and its cross-linking results in TatC dimers. **(A)** Immunoblot detection of Bpa photo cross-links in a strain producing TatABC_I50Bpa_. Irradiation by UV light results in formation of a ~55 kDa TatC homodimer (*) that is detected in the TatC blot (right) but not in the TatB blot (left). **(B)** Model of *E. coli* TatC. I50 of TatC is highlighted in red and is located at the end of TM1 (green) in transition to a less structured region of the first periplasmic loop (orange). The model is based on the *Aquifex aeolicus* TatC structure (PDB 4HTS; Ramasamy et al., 2013) and was modeled using RaptorX (Peng and Xu, 2011; Källberg et al., 2014). Molecular graphics and analyses were performed using UCSF Chimera (Pettersen et al., 2004). **(C)** Analysis of photo cross-links with Bpa exchanges at indicated TatC positions in the vicinity of I50. The position of the cross-link is indicated by an asterisk. SDS-solubilized membranes were used for the detection of Tat components as described previously (Taubert et al., 2015). **(D,E)** Activity of TatABC_I50Bpa_ as monitored by complementation of the SDS sensitivity **(D)** and chain formation phenotypes **(E)** of Tat deficient strains. SDS sensitivity results in lowered cell density, which is given as normalized OD_600_, the quotient of OD_600_ after 3 h of growth in 4% SDS/OD after 3 h of growth in 0% SDS. As control for these phenotypes, the same system was analyzed with a mutated Tat substrate-binding site. The mutations F94Q/E103A completely inactivate Tat systems (Huang et al., 2017, and this study).

In the course of *in vivo* photo-cross-linking analyses of TatC interactions with single TatC amino acids exchanged to *p*-benzoyl-L-phenylalanine (Bpa), we identified that a TatC_I50Bpa_ construct efficiently cross-linked to other TatC protomers, giving rise to a strong UV-induced shifted TatC band at ~55 kDa (Figure 1A, compare −UV/+UV). TatB is known to tightly interact with TatC, and TatB-TatC cross-links would have resulted in shifts of similar size, but as no TatB was detectable in these bands, it is likely that the shifted bands represented TatC-TatC cross-links. TatC is only present in oligomeric complexes in *E. coli*, and the position of the I50Bpa exchange must be therefore in close proximity to neighboring TatC protomers within Tat complexes. Within the first periplasmic loop (PPL1) of TatC, Ile50 is located in an α-helix oriented almost parallel to the surface of the membrane directly following TM1 (Figure 1B). Previous studies showed that other residues located in this region are crucial for Tat complex assembly or functionality (Allen et al., 2002; Barrett et al., 2005; Kneuper et al., 2012). Already in early studies, an exchange of the close-by residue Pro48 to Ala has been shown to inactivate the Tat system, which has been explained by a destabilization of protomer interactions within the complex (Allen et al., 2002; Behrendt and Brüser, 2014). While Pro48 is directed inward and thus likely positions the helix at its place, Ile50 is directed outward, which likely enables the contacts to neighboring TatC subunits. To examine potential TatC cross-links with Bpa exchanges in the vicinity of Ile50, we included cross-link analyses of TatABC systems with TatC exchanges A47Bpa, P48Bpa, L49Bpa, K51Bpa, and Q52Bpa (Figure 1C). In case of P48Bpa and K51Bpa, the abundance of TatC was reduced, most likely due to destabilization and degradation effects. A cross-link was also observed with L49Bpa, but I50Bpa gave the strongest cross-link. In further analyses, we therefore concentrated on I50Bpa. The TatABC_I50Bpa_ system was active, as shown by the complementation of SDS sensitivity and chain formation phenotypes of a *tatABCDE* mutant strain (Figures 1D,E).

### *p*-Benzoylphenylalanine or Tyrosine at Position I50 of TatC Influence the Equilibrium Between Three Assembly States of Twin-Arginine Translocation Complexes

**Figure 2:**
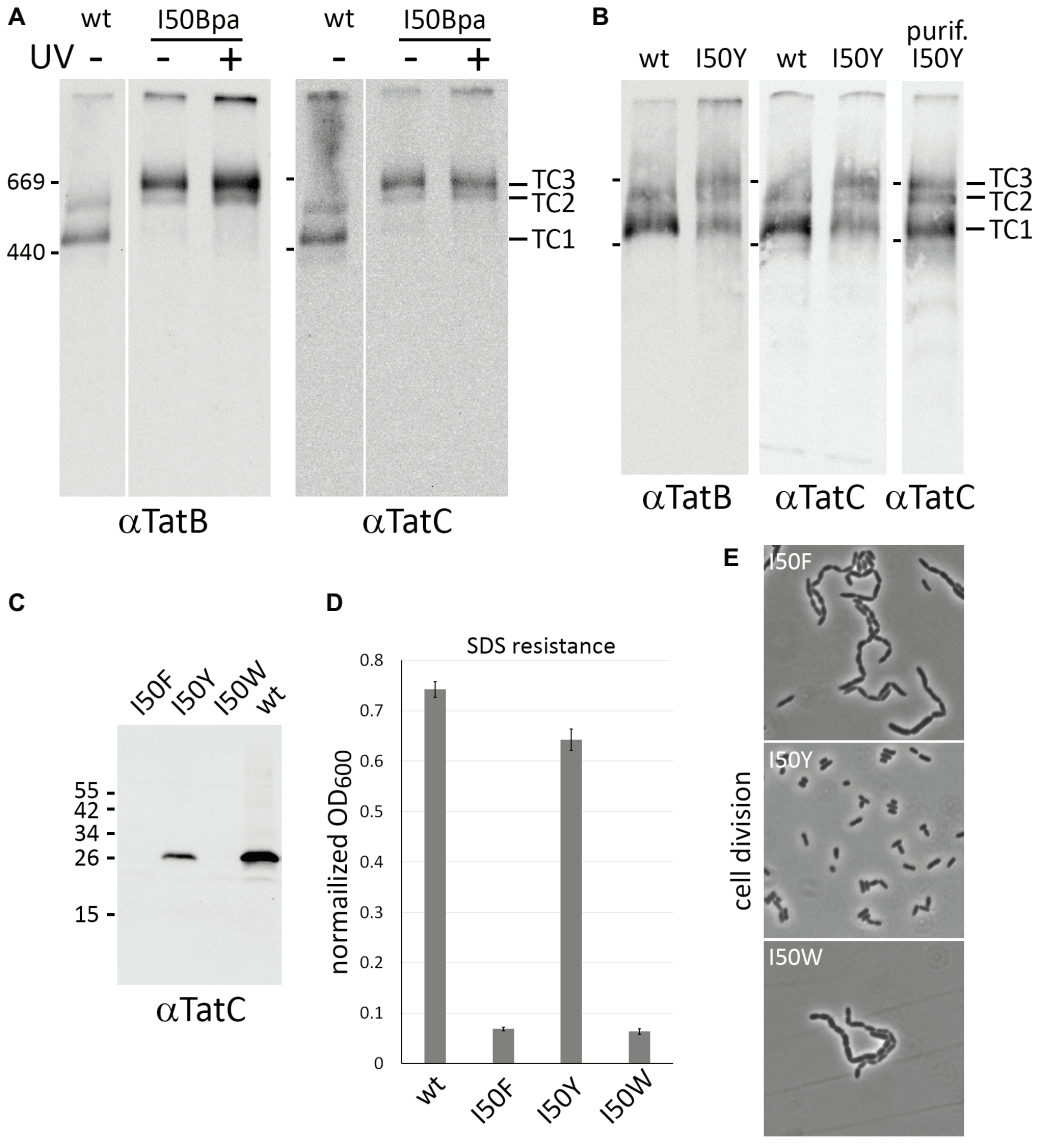
An equilibrium between three Tat complexes is influenced by Bpa or Tyr exchanges at position I50. **(A)** BN-PAGE/Western-blot analysis of Tat complexes formed by wild-type TatABC and TatABC_I50Bpa_ systems. As indicated, blots were developed using TatB or TatC specific antibodies. Positions of marker proteins are indicated on the left of the blots, positions of the three Tat complexes are indicated on the right. White lines separate lanes from one blot that were not directly neighboring. **(B)** BN-PAGE/Western-blot analysis of Tat complexes formed by wild-type TatABC and by TatABC_I50Y_ systems. The Tat complexes of the latter were also enriched by affinity chromatography (lane purif. I50Y). **(C)** SDS-PAGE/Western-blot analysis of TatC with I50 exchanged by indicated aromatic natural amino acids. TatC from the non-mutated system is analyzed for comparison. **(D,E)** Activity of Tat systems with wild-type TatC or indicated TatC variants, as monitored by complementation of the SDS sensitivity **(D)** and chain formation phenotype **(E)** of Tat deficient strains. The SDS sensitivity is reflected by the normalized OD_600_ as described in Figure 1.

Previous studies had already indicated a strong influence of P48, which is located only half a helical turn away from I50, on the stability of TatBC complexes (Allen et al., 2002; Barrett et al., 2005; Kneuper et al., 2012; Behrendt and Brüser, 2014). To our knowledge, mutations of I50 have never been analyzed. To address complex stability of Tat systems containing I50Bpa, membranes were solubilized with 1% (w/v) digitonin and subjected to BN-PAGE/Western-blot analysis (Figure 2). While wild-type TatABC produced the two known TatB- and TatC-containing complexes (TC1 and TC2), TatABC_I50Bpa_ predominantly formed a new complex that was clearly shifted to a higher molecular mass and also contained TatB and TatC (Figure 2A). In addition, a less dominant complex with the size of TC2 or TC2S of the wild-type Tat system was observed, which will be assigned later (see below). The shifted complex did not result from UV-cross-linking, as the pattern did not change without UV illumination. This suggested that most likely a non-covalent interaction of the aromatic benzophenone side chain was causing the shift. To analyze whether aromatic side chains of natural amino acids can cause the same shift, we substituted Ile50 by tyrosine, tryptophan, or phenylalanine. Before analyzing the corresponding Tat complexes, we examined the activity and formation of the mutated components (Figures 2C–E). The I50W and I50F substitutions resulted in inactivation and absence of TatC, and thus, most likely caused a complete degradation of TatC. Notably, an I50Y substitution lowered the abundance of TatC but did not inactivate the system. The data underlined the importance of that position for the structural integrity of the Tat system. The active TatABC_I50Y_ system was then analyzed by BN-PAGE (Figure 2B). The usual complexes TC1 and TC2 were formed that can be also observed by non-mutated Tat systems, but there was clearly the additional band of the shifted complex (TC3) that had been previously observed with TatABC_I50Bpa_. Together, these analyses indicated that an equilibrium exists between three Tat complexes in active Tat systems, and this equilibrium is strongly influenced by I50 exchanges to either Bpa or Tyr, which introduces benzophenone or hydroxyphenol moieties, respectively. Both side chains allow aromatic contacts as well as hydrogen bonding (mediated by the keto group of benzophenone or the hydroxy group in tyrosine), and this combination is likely responsible for the stabilization of TC3, which apparently otherwise is likely only a short-lived complex. As cross-links were irrelevant for TC3-formation, we did not apply cross-linking for all further analyses with Bpa-containing constructs.

### TC3 Requires TatA to Be Formed

**Figure 3:**
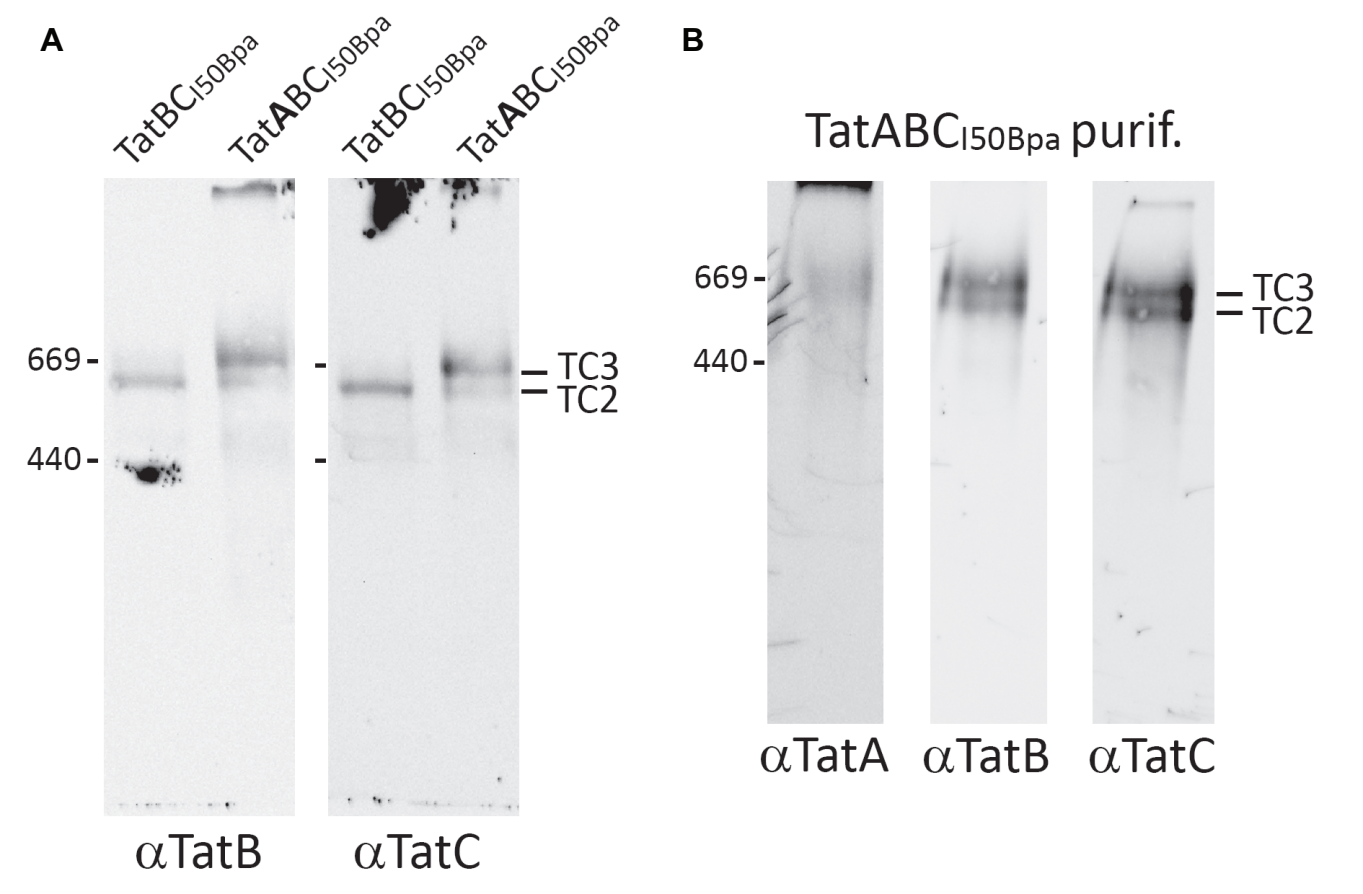
TatA is required for the formation of TC3. **(A)** BN-PAGE/Western-blotting detection of Tat complexes in the absence or presence of TatA. Analyses of Tat complexes in strains producing TatABC_I50Bpa_ or TatBC_I50Bpa_. **(B)** Detection of the three Tat components within TC3 after a two-step purification (Ni-affinity purification and size exclusion chromatography using a Superose 6 column). Positions of TC2 and TC3 are indicated on the right, size markers on the left, and the used antibodies below the corresponding blots.

A reason for the I50Bpa-induced stabilization of a Tat complex at a higher molecular mass could have been an enhanced affinity of TatA to TatBC. A reduced TatA-dissociation would explain the observed depletion of TC1. We therefore addressed this potential role of TatA by comparing the BN-PAGE detectable complexes formed in the presence or absence of TatA (Figure 3). The absence of TatA resulted in the formation of a complex of the size of TC2 or TC2S, which would need a substrate detection for clear differentiation (see below), but TC3 was clearly absent. Notably, the TatBC_I50Bpa_ system did not result in TC1, which is the complex usually formed in the absence of TatA (Behrendt and Brüser, 2014), indicating that the I50Bpa mutation in TatC also results in an increased affinity of TatB to TatA-binding sites. It is established that TatB can in principle bind to TatA-binding sites in active Tat systems (Habersetzer et al., 2017), and apparently, the I50Bpa mutation enhances this interaction to an extent that no TC1 is detected anymore. The data also clearly show that TatA is required for the formation of TC3 in TatABC_I50Bpa_ systems, implying that TatA was likely a constituent of the new complex. To address this aspect directly, we purified TatC-h6-tagged Tat complexes in a strain producing the TatABC_I50Bpa_ components, using a combination of immobilized metal affinity chromatography (IMAC) and size exclusion chromatography (SEC). Thus, the enriched complexes were analyzed by BN-PAGE/Western-blotting (Figure 3). Non-bound TatA, which is known to form homooligomeric assemblies that are detected as a “ladder” over a broad range of sizes in BN-PAGE (Barrett et al., 2005; Richter and Brüser, 2005; Behrendt et al., 2007), was efficiently removed by the purification. We now were able to detect TatA in a weak band of the size of the two TatBC-containing complexes, likely indicating the presence of TatA in these complexes. Based on the TatA dependence of TC3 in conjunction with the detection of TatA in a BN-PAGE band of that size, we suggest that both detected complexes contain significant amounts of TatA, which likely explains the shift of these complexes in comparison with TC1. Consequently, the I50Bpa exchange likely enhanced the stability of a TatA-assembled state of the Tat system.

### Substrate Is Likely Already Transported by the Detected TC3

**Figure 4:**
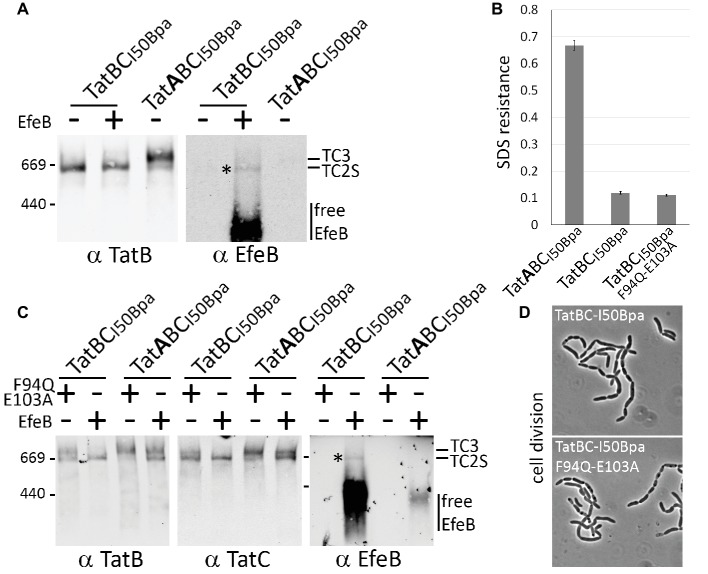
A mutated substrate-binding site can result in TC3 formation in the absence of TatA. **(A)** Detection of recombinant Tat substrate EfeB in TC2. Overproduction of EfeB leads to the detectability of this Tat substrate in TC2 formed by TatBC_I50Bpa_. After development of the blot using antibodies directed against TatB (left blot), the blot was stripped (see methods) and developed again with antibodies recognizing EfeB (right blot). **(B,D)** The absence of TatA inactivates the Tat system also in case of TatBC_I50Bpa_. As monitored by SDS resistance **(B)** and the cell division **(D)**, TatBC_I50Bpa_ does not complement the deficiency phenotypes of the *E. coli* Tat mutant strain DADE. The SDS sensitivity is reflected by the normalized OD_600_ as described in Figure 1. We used the TatABC_I50Bpa_ as positive control, which behaved like the wild type (see Figure 1D or 2D). **(C)** Mutational inactivation of the twin-arginine motif-binding site in TatC influences the formation of TC2 and TC3. Comparison of systems with inactivated Tat substrate-binding sites (F94Q, E103A) with systems in which the Tat substrate EfeB has been overproduced. After development of the blot using TatC antibodies, the blot was stripped and developed using antibodies recognizing EfeB. Note that EfeB is not detected in the TC2 formed by the active TatABC_I50Bpa_ system, most likely due to active transport that is likely the reason for the lowered abundance of EfeB in the membranes.

To clearly differentiate, whether the detected TatBC_I50Bpa_ complex that is formed in the absence of TatA contains substrate or not, we examined the complex after recombinant production of the *E. coli* Tat substrate EfeB (formerly known as YcdB). EfeB has been shown to bind Tat complexes with sufficient affinity to detect the interaction by BN-PAGE/Western-blot analysis (Behrendt and Brüser, 2014). EfeB could be clearly detected in the TatBC_I50Bpa_ complex, indicating that it represented the substrate-bound TC2S (Figure 4A). Note that, due to the lack of TatA, the TatBC_I50Bpa_ system is inactive (Figure 4B), and EfeB accumulates strongly in the membranes, whereas the active transport by the TatABC_I50Bpa_ system markedly reduces the abundance of EfeB in the membranes (Figure 4C, compare free EfeB signals, lanes 2 and 4). The TC2 complex was also present in TatABC_I50Bpa_ systems, migrating as a second band below TC3, but the complex was less abundant, and no substrate was detectable. Also TC3 of the TatABC_I50Bpa_ system contained no detectable substrate, although it was more abundant than TC2S of the TatBC_I50Bpa_ system (Figure 4C), in line with the generally accepted idea that transport occurs upon TatA recruitment. The detected TC3 therefore likely represents a stabilized active state of the Tat system in which the complexes have already accomplished transport.

To examine whether TC3 depends also on substrate binding, we analyzed the TatABC_I50Bpa_ system with an inactivated twin-arginine motif-binding site, achieved by a F94Q/E103A double exchange in TatC. The inactivity of that system has been previously established by others (Huang et al., 2017) and was confirmed in our hands (see negative control in Figures 1D,E). BN-PAGE analyses demonstrated that the inactivation of the substrate-binding site did not diminish TC3, clearly demonstrating that substrate binding was not a prerequisite for the shift (Figure 4C). When we introduced the same inactive substrate-binding site into the TatBC_I50Bpa_ system, we found in analogous BN-PAGE/Western-blotting analyses that a small portion of the TatBC_I50Bpa/F94Q/E103A_ complex had shifted to TC3, which is normally only found in the presence of TatA (Figure 4C). Apparently, TatB associated with TatBC_I50Bpa/F94Q/E103A_ just like TatA to TatBC_I50Bpa_. In conclusion, the F94Q/E103A mutations of the substrate-binding site caused a relaxed, not TatA or TatB differentiating enhanced association of TatA or TatB to TatC, leading to increased amounts of the TC3 with TatABC_I50Bpa/F94Q/E103A_ systems and even a TC3 in TatBC_I50Bpa/F94Q/E103A_ systems.

## Discussion

In this study, we analyzed in some detail a third Tat complex, termed TC3, that became the dominant Tat complex if only one TatC position, I50, was mutated to the artificial aromatic amino acid Bpa. We could demonstrate that side chain properties and not a photo activatable cross-link were the basis for the detection of this complex. The complex was active, suggesting that it likely represents a naturally occurring association of Tat components that is stabilized by the amino acid exchange (Figure 1). As additional evidence for this, we found that the exchange of I50 by the natural amino acid Tyr could stabilize TC3, too. In case of this I50Y exchange, which was active as well, all three Tat complexes were clearly detectable (Figure 2), suggesting that there is an equilibrium between Tat associations that can be influenced by single amino acid exchanges at position I50. The fact that the I50Bpa exchange can be photo cross-linked to neighboring TatC subunits supports the idea that certain TatC-TatC interactions stabilize the association that is detected as TC3. If there is an equilibrium between TC1, TC2, and TC3, this means that either TC3 is a lowly populated transiently formed complex in the wild-type system or it disassembles upon solubilization to TC2 or TC1, if it is not stabilized. The fact that the complex depends on TatA and that TatA can be co-purified with that complex (Figure 3) suggests that TC3 may be the active translocon association. In agreement with the idea that TC3 transports and therefore releases Tat substrates, we found that this complex did not contain any detectable Tat substrate (Figure 4). In the absence of TatA, when no transport can take place, a TC2S is formed. However, a TC2 band that is detected below the TC3 band in the active TatABC_I50BPA_ system did not contain any detectable substrate. This indicates that either TC2 with or without substrate cannot be differentiated by BN-PAGE migration behavior with I50Bpa TatC variants, or substrate does not accumulate to a detectable extent at TC2 in active (TatA-containing) systems (Figure 4). The detection of translocon bound substrate in TC2 of TatBC_I50BPA_ systems may thus be facilitated by the accumulation of substrates due to the absence of TatA, which is evidenced by the large amounts of EfeB in the membranes of that strain.

An older study questioned the physiological relevance of the Tat complexes that are detected by BN-PAGE analyses (Barrett et al., 2007). The authors used mutated TatA that significantly improved Tat functionality in systems lacking TatB (Blaudeck et al., 2005). However, the mutations were in the N-terminus of TatA and cannot be expected to influence the low detergent resistance of the TatA-TatC interaction, which is mediated mainly by the trans-membrane domain (Habersetzer et al., 2017). It is well known that *E. coli* TatA and TatC catalyze translocation with extremely low efficiency (Ize et al., 2002; Blaudeck et al., 2005). Although no easily detectable detergent-stable TatAC complexes are formed, TatA depletes TatC-only complexes in the absence of TatB, indicating that the TatA-TatC interaction engages most TatC *in vivo* and thereby does not permit the formation of TatC-only complexes (Behrendt et al., 2007). The interaction is also supported by *in vitro* TatAC cross-links in the absence of TatB (Blümmel et al., 2015). The absence of BN-PAGE-detectable detergent-solubilized TatAC complexes in systems lacking TatB therefore should not be taken as argument against the relevance of identified Tat complexes in TatABC systems. TC1 and TC2 can bind substrates, resulting in TC1S and TC2S (Behrendt and Brüser, 2014). Substrates were bound *in vivo*, supporting the physiological relevance of these complexes. Now we found a third complex (TC3) that became detectable due to stabilizing interactions of altered side chains. We propose that this is the association that actually delivered the substrate across the membrane.

**Figure 5:**
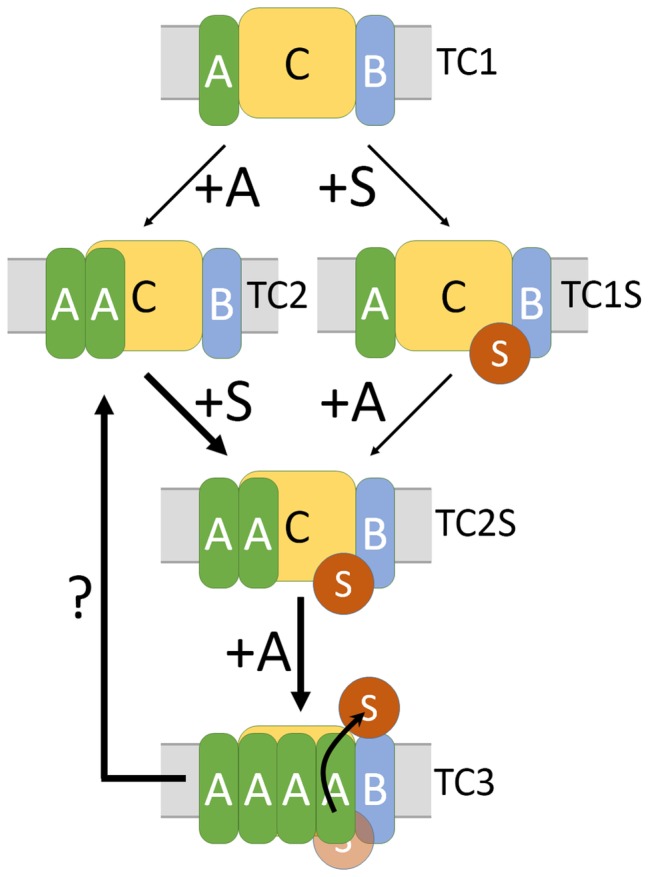
Integrative model for the assembly of active TatABC complexes. Based on the data of this study, in combination with the published work, we propose the following assembly model for active Tat complexes: TC1 represents a Tat complex that can in principle be formed in the absence of TatA and likely is the core complex. TC2 is less stable in detergent solution but already present in wild-type TatABC systems. Both, TC1 and TC2 can in principle bind Tat substrates. Substrate-bound TC2 (TC2S) can switch to TC3, which has an increased affinity to TatA. When TC3 is formed, the substrate can be translocated through the membrane, most likely in an TatA-influenced environment. In the model, “+A” at arrows indicates that more detergent-resistant TatA is bound. “+S” indicates the binding of the twin-arginine motif to its binding site at the TatBC core.

The knowledge about the three *E. coli* Tat complexes detected so far can be summarized in the following assembly pathway model (Figure 5): TC1 can be formed without TatA and may be regarded as the core unit. TC2 is detectable when TatA is present in the system and we think that this likely represents the resting state (Behrendt and Brüser, 2014). Harsh solubilization conditions deplete TC2 in favor of TC1. When TatB is recombinantly overproduced, complexes of the size of TC2 can also be formed without TatA in the system (Behrendt et al., 2007), which agrees with the finding that TatB can occupy TatA-binding sites at TatC under certain conditions (Habersetzer et al., 2017). As both, TC1 and TC2, can bind Tat substrates (Behrendt and Brüser, 2014), it is unclear whether TatA is recruited to TatBC upon substrate binding or whether substrates are recruited to TC2 that already contains TatA. Both scenarios can explain the previously observed co-localization of TatA-XFP fusion proteins with TatBC upon substrate overproduction (Alcock et al., 2013; Rose et al., 2013). A stabilization of the TatA-TatBC interaction by substrate binding may compensate for a destabilization that can result from the XFP fusion to the tightly interacting smaller TatA protomers, which would also explain the observations that have been interpreted as substrate-induced assembly. Substrate binding clearly influences the interaction of TatBC with TatA, which can be monitored by cross-linking (Dabney-Smith et al., 2006), but TatA is always directly or indirectly associated with TatBC also under resting conditions (Aldridge et al., 2014; Behrendt and Brüser, 2014). The TatA-dependent rearrangement or conformational transition within Tat complexes likely paves the way for Tat transport. It could be demonstrated that a substrate interaction with TatA results in conformational transitions that destabilize the membrane, and this has been suggested to enable the translocation (Brüser and Sanders, 2003; Hou et al., 2018). All published data suggest that there exists a transiently enhanced affinity to TatA during transport, and it is a matter of kinetic stability, whether or not this active TatABC translocon can be detected. Our analyses now suggest that single amino acid exchanges at position I50, which is positioned at a TatC-TatC interface, can stabilize this active complex. This third Tat complex (TC3) likely switches back to the TC2 state when transport has taken place (question mark in Figure 5), and the kinetics of this transition is apparently slowed down by the amino acid exchange, thereby allowing the detection of TC3. The stabilization of TC3 does not require substrate binding and therefore is only a matter of TatABC interactions. As Bpa cross-linking indicates a close proximity of the I50 position to neighboring TatC protomers in the complex, a conformational transition that relies on TatC-TatC interactions is likely responsible for higher TatA affinity. Substrate binding may trigger the switch to this altered interaction in the native, non-mutated system, which explains why TC3 is normally very transient. The data that we obtained with TatBC systems indicate that the I50Bpa mutation enhances not only the TatA affinity but also the TatB affinity to TatC. In contrast to the TatABC_I50Bpa_ system, the TatBC_I50Bpa_ system is not active, indicating that TatB cannot substitute for TatA. However, an inactivated substrate-binding site can trigger the transformation of TC2 to TC3 even in the absence of TatA (Figure 4). The conformation of the substrate-binding site might therefore be important for a selective binding of TatA to this site, which is crucial for the transport process. In agreement with this observation, cross-linking studies with the plant TatABC system have shown in the past that TatA associates with the substrate-binding site (Aldridge et al., 2014).

## Data Availability

All datasets generated for this study are included in the manuscript.

## Author Contributions

HG obtained all presented data, except the analysis shown in Figure 1C, which was done by CN. DM-B and EH were involved in early stages of the project and did initial experiments. TB designed and supervised the study and wrote the manuscript together with HG. All authors contributed to the final manuscript.

### Conflict of Interest Statement

The authors declare that the research was conducted in the absence of any commercial or financial relationships that could be construed as a potential conflict of interest.
